# Cereblon regulates NK cell cytotoxicity and migration via Rac1 activation

**DOI:** 10.1002/eji.202149269

**Published:** 2021-09-18

**Authors:** Cinzia Fionda, Helena Stabile, Rosa Molfetta, Andrea Kosta, Giovanna Peruzzi, Silvia Ruggeri, Alessandra Zingoni, Cristina Capuano, Alessandra Soriani, Rossella Paolini, Angela Gismondi, Marco Cippitelli, Angela Santoni

**Affiliations:** ^1^ Department of Molecular Medicine, Laboratory affiliated to Istituto Pasteur‐Fondazione Cenci Bolognetti Sapienza University of Rome Italy; ^2^ Center for Life Nano‐ & Neuro‐Science Fondazione Istituto Italiano di Tecnologia (IIT) Rome Italy; ^3^ Department of Experimental Medicine Sapienza University of Rome Rome Italy; ^4^ IRCCS Neuromed Pozzilli IS Italy

**Keywords:** Cereblon, E3 ubiquitin ligase, Natural killer cells, Lenalidomide, Rac1

## Abstract

Rearrangement of the actin cytoskeleton is critical for cytotoxic and immunoregulatory functions as well as migration of natural killer (NK) cells. However, dynamic reorganization of actin is a complex process, which remains largely unknown. Here, we investigated the role of the protein Cereblon (CRBN), an E3 ubiquitin ligase complex co‐receptor and the primary target of the immunomodulatory drugs, in NK cells. We observed that CRBN partially colocalizes with F‐actin in chemokine‐treated NK cells and is recruited to the immunological synapse, thus suggesting a role for this protein in cytoskeleton reorganization. Accordingly, silencing of CRBN in NK cells results in a reduced cytotoxicity that correlates with a defect in conjugate and lytic synapse formation. Moreover, CRBN depletion significantly impairs the ability of NK cells to migrate and reduces the enhancing effect of lenalidomide on NK cell migration. Finally, we provided evidence that CRBN is required for activation of the small GTPase Rac1, a critical mediator of cytoskeleton dynamics. Indeed, in CRBN‐depleted NK cells, chemokine‐mediated or target cell–mediated Rac1 activation is significantly reduced. Altogether our data identify a critical role for CRBN in regulating NK cell functions and suggest that this protein may mediate the stimulatory effect of lenalidomide on NK cells.

## Graphical abstract

Cereblon (CRBN), the primary target of the Immunomodulatory drugs (IMiDs), regulates the activation of the small GTPase protein Rac1 and is required for NK cell migration and cytotoxicity. Our study reveals new information on complex mechanisms underlying cytoskeleton reorganization and stimulatory effects of IMiDs in NK cells.

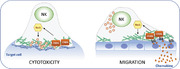

## Introduction

Natural Killer (NK) cells are innate lymphoid cells that play an important role in immune response against infection diseases and cancer, via contact‐dependent cellular cytotoxicity and cytokine and chemokine production [1, 2].

NK cell cytotoxicity is a multistep and tightly controlled process. Following NK cell activating receptor engagement, an early step is NK cell conjugation with the target cell followed by formation of the immunological synapse (IS) at the contact site, lytic granule polarization, and degranulation. Cell adhesion molecules, in particular the integrin lymphocyte function‐associated antigen‐1 (LFA‐1), play an essential role in these mechanisms. The engagement of LFA‐1 with its ligand ICAM‐1 mediates the firm adhesion to the target cell and drives the accumulation of filamentous actin (F‐actin) as well as lytic granule convergence toward the microtubule organizing center (MTOC) and polarization at IS [3, 4]. Moreover, it is well established that cytoskeleton reorganization is necessary for the formation of IS and cytotoxic function [5, 6].

Adhesion molecules, chemokines, and cytoskeleton rearrangement are also important regulators of NK cell migration. In particular, the binding of chemokines to their receptors triggers a complex signaling cascade that supports leukocyte migration, regulates the integrin adhesiveness, and causes a fine remodeling of cytoskeleton components. Key regulators of NK cell cytotoxicity and migration are the RhoA, Rac1, and Cdc42 small GTPases. In migrating cells, Cdc42 and Rac1 cause actin polymerization leading to the formation of filopodia and lamellipodia, respectively [7, 8]. Moreover, as signaling molecules downstream of integrin and NK activating receptors, these proteins are required for the assembly of IS and MTOC polarization [9, 10, 11].

Several studies described the capability of immunomodulatory drugs (IMiDs, thalidomide, lenalidomide and pomalidomide) to regulate NK cell function by stimulating the expression of different activating receptors [12], cytokine production [13], NK cell proliferation, and natural and antibody‐dependent cytotoxicity [14, 15, 16]. Moreover, lenalidomide can directly lower the threshold for NK cell activation, by augmenting rearrangements in cortical actin at the human NK cell IS, but the molecular events involved were not elucidated [15].

A key target of these drugs is the protein cereblon (CRBN) [17], even if CRBN‐independent effects of lenalidomide have been described [18]. CRBN is the receptor substrate of the cullin‐4‐RING ubiquitin ligase (CLR4^CRBN^) complex, containing also cullin‐4 (CUL4), the DNA damage binding protein‐1 (DDB1), and the RING‐finger protein (ROC1). Moreover, CRBN can function in a ubiquitin‐independent manner by binding and modulating the membrane expression and/or the function of different proteins [19]. Through their glutarimide ring, IMiDs can interact with CRBN, thus altering the substrate specificity of CLR4^CRBN^ complex [20, 21] or disrupting CRBN binding to other proteins [19]. IMiDs can differently regulate the ubiquitination and degradation of CRBN substrates [22]. Indeed, IMiDs can disturb the interaction of CRBN with the transcription factor MEIS2 by preventing its degradation, but they enhance the CRBN‐mediated recruitment and ubiquitination of IKZF1 or IKZF3 (where IKZF is Ikaros transcription factor) proteins [23].

In the context of immune cells, CRBN was shown to limit murine CD4^+^ T cell activation via epigenetic repression of the Kv1.3 potassium channel expression required for calcium influx [24], and to mediate human T cell co‐stimulation by lenalidomide and pomalidomide causing IKZF1 and IKZF3 degradation [25]. Moreover, CRBN functions to harness antigen‐specific CD8^+^ T‐cell effector responses through c‐myc‐dependent regulation of the central metabolism [26]. A study recently reported that IMiDs‐treated human NK cells have increased granzyme B (GZM‐B) expression and cytotoxicity via Zap‐70 activation and CRBN‐degradation of IKZF3 [16].

In this study, we show that CRBN is required for NK cell migration and cytotoxicity. We demonstrate for the first time that CRBN partially localizes with accumulated F‐actin in chemokine‐treated NK cells or at IS and can regulate Rac1 activation. Taken together, these observations indicate a role for CRBN in the regulation of key NK cell functions and suggest that lenalidomide‐mediated alteration of CRBN activity is responsible for its stimulatory effect on NK cells.

## Results

### The protein CRBN co‐localizes with F‐actin in NK cells

**Figure 1:**
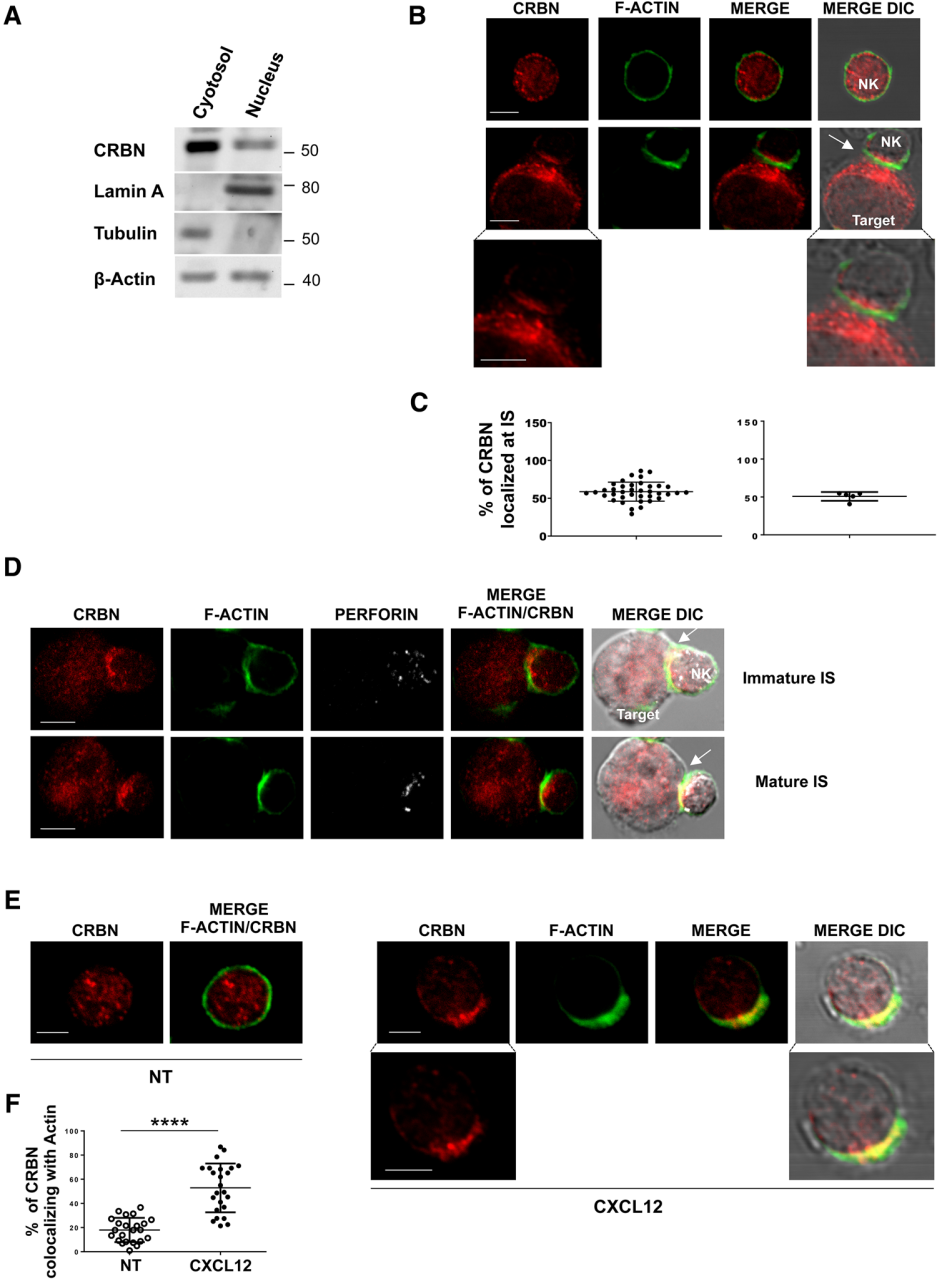
Cytoskeleton remodeling causes a cellular redistribution of CRBN in NK cells. (A) Immunoblot analysis of cytosolic and nuclear extracts obtained from primary NK cells with anti‐CRBN, anti‐Lamin A (as a nuclear marker), and anti‐tubulin (as a cytosolic marker) antibodies. β‐Actin was used as loading control. The blot shown is representative of three independent experiments. Original blot picture in Supporting Information 8A. (B and D) Primary NK cells alone or after incubation with K562 target cells were stained with CRBN (red), phalloidin (green) (B), and perforin (white). Representative images of conjugates are shown as a single optical section. (C) Percentage of CRBN localized at the site of contact with K562 out of the total present in NK cells. Representative data of 30 conjugates collected from a single experiment (left panel) and the mean ± SD of five independent experiments are shown (right panel). (E) Primary NK cells were allowed to adhere in the presence or absence of 10 nM CXCL12 and stained with CRBN (red) and phalloidin (green). Representative images of three experiments are shown. Fluorescence images shown in (B) and (E) were acquired using 60×/1.35 NA objective, zoom 6 (800 × 800 pixel). Bottom panels represent the magnification of the corresponding images acquired with zoom 18 (1600 × 1600 pixel). Scale bars represent 5 μm. DIC, differential interference contrast. (F) Percentage of CRBN intensity colocalizing with actin out of the total. A representative of three experiments with 25 cells for each condition is shown (****p < 0.0001, paired Student's *t*‐test). Data show mean ± SD.

Little evidence has been provided so far on the role of protein CRBN in NK cells [16]. By western blot assay, we initially demonstrated that CRBN is expressed at protein level in these lymphocytes both in cytosolic and nuclear compartment (Fig. 1A). Then, we analyzed CRBN cellular distribution in NK cells contacting target cells during the formation of IS. IL‐2‐activated NK cells and NK‐92 cell line were incubated with K562 susceptible target cells and stained for CRBN, F‐actin, and perforin (as a marker of lytic granules). When visualized using confocal fluorescent microscopy, CRBN was found to accumulate at the interface with target cells (Fig. 1B and C and Supporting Information 1A) during the formation of mature NK cell IS, which was identified by the accumulation of F‐actin and the polarization of lytic granules [27]. Notably, the recruitment of CRBN precedes granule polarization; indeed, CRBN is re‐localized at the interface with target cells also in the immature synapse (IS with F‐actin accumulation without perforin polarization) (Fig. 1D and Supporting Information 1B). These findings suggested that, in NK cells contacting target cells, cellular relocation of CRBN could be linked to actin polymerization driving the IS assembly.

On the basis of these observations, we examined whether a different stimulus known to induce cytoskeleton reorganization, such as a chemokine, could affect CRBN cellular distribution. We observed that CRBN localized to the lamellipodial leading edge and partially overlapped with F‐actin in CXCL12‐treated NK cells (Fig. 1E).

These findings indicate that stimuli known to induce cytoskeleton remodeling can also cause a cellular redistribution of CRBN in NK cells.

### The protein CRBN regulates NK cell cytotoxicity

**Figure 2:**
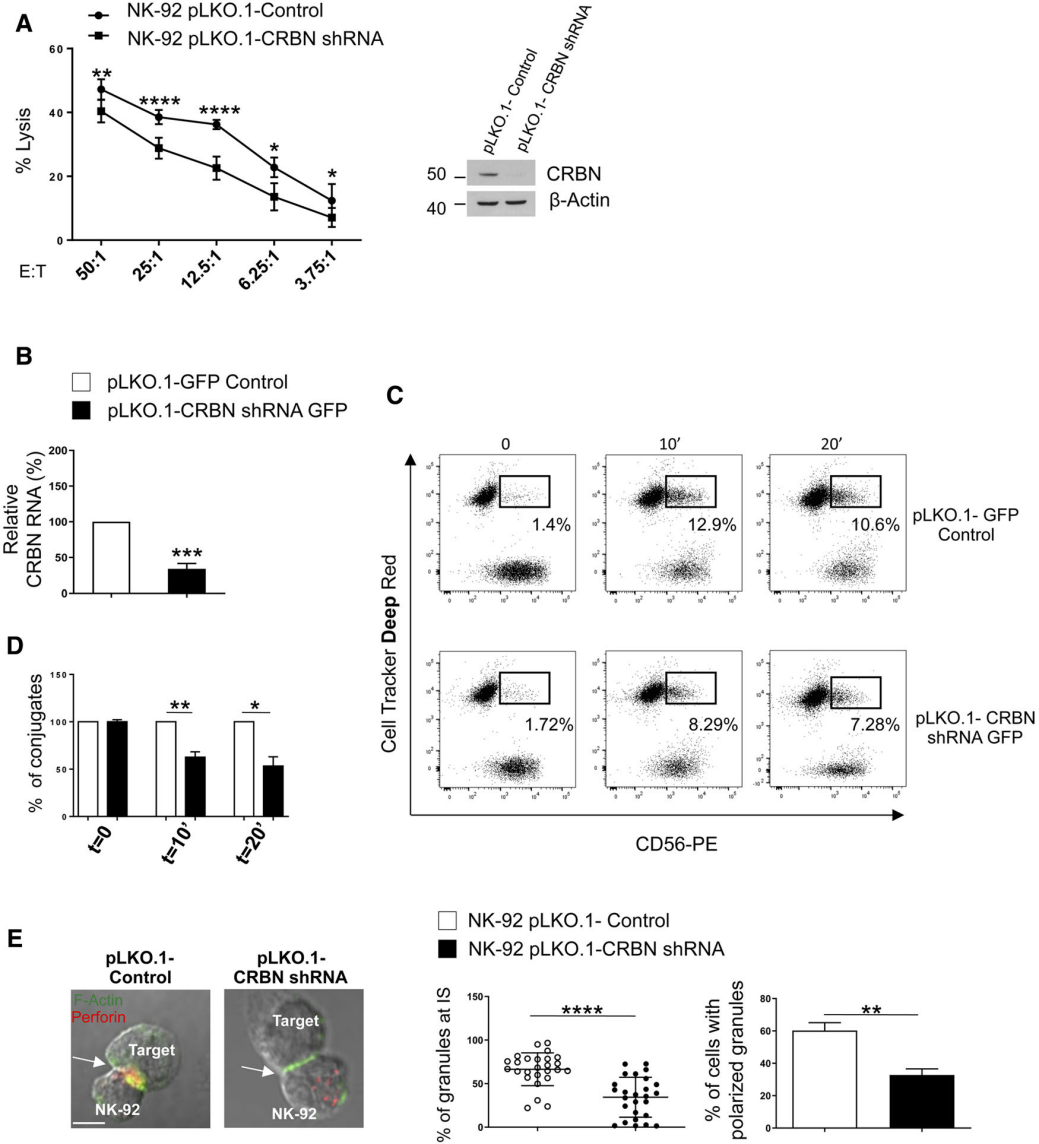
Silencing of CRBN impairs NK cell cytotoxicity. (A) Immunoblot analysis of CRBN and β‐actin (used as loading control) levels in NK‐92 cells transduced with lentivirus pLKO.1‐shRNA‐CRBN or nontarget shRNA (right panel). The blot shown is representative of three independent experiments. Original blot picture in Supporting Information 8B. Cytotoxicity of control or CRBN‐depleted NK‐92 cells were tested against K562 target cells by ^51^Cr‐release assay (left panel). Data show mean ± SD from three independent experiments. All experimental groups were analyzed in technical triplicates for each condition per experiment (****p < 0.0002; **p < 0. 005; *p < 0. 05, paired Student's *t*‐test). (B) Primary NK cells transduced with lentivirus pLKO.1‐shRNA‐CRBN‐GFP or nontarget shRNA were sorted (see gating strategy described Supporting Information 2A) and analyzed for CRBN mRNA expression by real‐time qRT‐PCR. Data were normalized with GAPDH and referred to the cells infected with nontarget shRNA, considered as calibrator. Mean ± SD of three experiments in which samples were analyzed in technical triplicates for each condition (***p < 0.0002, paired Student's *t*‐test). (C) Infected NK cells were incubated with Cell Tracker Deep Red‐loaded K562 target cells for the indicated time. After fixation, cells were stained with anti‐CD56‐PE mAb and the percentage of effector‐target conjugates were analyzed by flow cytometry (see gating strategy described Supporting Information 2B). (D) Histogram represents the percentage of NK‐K562 conjugates. Data show mean ± SD of four independent experiments with duplicate samples for each condition per experiment (**p < 0.005; *p < 0.05, paired Student's *t*‐test). (E) Control‐ or CRBN shRNA NK‐92 cells were incubated with K562 target cells and stained with CRBN (red), phalloidin (green), (B) and perforin (white). Scale bar represents 5μm. A representative image is shown (left panel). The percentage of perforin fluorescence at IS of a representative experiment and the mean percentage of conjugates with polarized granules ± SD of *n* ≥ 30 for each cell type of three independent experiments are shown (****p < 0.0001; **p < 0.005, paired Student's *t*‐test) (right panel).

Polarization of actin cytoskeleton at the IS is a crucial event for NK cell cytotoxicity [5, 6, 9, 28]. Since CRBN localizes at the IS, we hypothesized a possible effect of this protein on early steps of NK cell lysis. To verify this possibility, we stably expressed CRBN shRNA or shRNA scramble in the NK‐92 cell line. Then, we evaluated the effect of CRBN knockdown (Fig. 2A, right panel) on the capability of NK‐92 cells to kill K562 target cells in a standard ^51^Cr‐release assay. We observed that loss of CRBN expression resulted in a significant reduction in NK cell cytotoxicity relative to the scramble control (Fig. 2A, left panel).

As an early step of NK cell cytotoxicity is the binding to target cells, we evaluated if CRBN depletion could hinder cell killing by perturbing conjugate formation. Control‐infected and CRBN silenced primary NK cells and Cell Tracker Deep Red‐loaded K562 target cells were co‐incubated at 37°C for different lengths of time, stained with anti‐CD56/PE, and the percentage of conjugates was determined using flow cytometry (Supporting Information 2B). As shown in Fig. 2B–D, we found a significant lower percentage of conjugates after CRBN knockdown. Similar results were obtained in both CRBN‐silenced NK‐92 cells (Supporting Information 2C) and primary NK cells treated with Homo‐Protac CRBN degrader 1 (PROTAC‐CRBN), a compound able to induce potent CRBN degradation (Supporting Information 3A and B) [29]. In these cells, we also observed a lower percentage of mature synapses as compared with control cells by fluorescence microscopy (Fig. 2E and Supporting Information 3C).

CRBN regulates NK cell cytotoxicity via IKZF3‐mediated regulation of GZM‐B expression [16]. However, our data indicate that loss of CRBN did not affect IKZF1 or IKZF3 expression as well as GZM‐B mRNA and protein levels (Supporting Information 4A–F). Based on these observations, we could exclude the involvement of these proteins in the regulation of NK cell cytotoxicity by CRBN.

Together, these results indicate that CRBN is recruited at NK cell IS, is involved in conjugate formation and in granule polarization, and regulates NK cell–mediated cytotoxicity.

### Regulation of NK cell migration by CRBN

**Figure 3:**
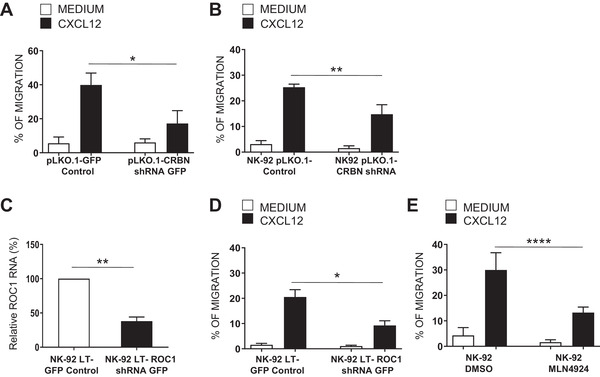
Regulation of NK cell migration by E3 ligase activity of CRBN. Primary NK cells infected with lentivirus pLKO.1‐shRNA‐CRBN‐GFP (A) and NK‐92 cell line upon transduction with lentivirus pLKO.1‐shRNA‐CRBN (B) or LT‐shRNA‐ROC1 (D) or upon treatment with MLN4924 (1 μM) for 18 h (E) were assayed for their ability to migrate toward a 10 nM CXCL12 gradient. The mean ± SD of the percentage of migrated cells obtained from at least three independent experiments is shown with duplicate samples for each condition per experiment (*p < 0.05; **p < 0.005; ****p < 0.0001 paired Student's *t*‐test). (C) Total mRNA obtained from sorted GFP^+^ NK92 cells infected with lentivirus LT or LT‐shRNA‐ROC1 was analyzed for ROC1 mRNA expression by qRT‐PCR. Data were normalized with GAPDH and referred to the cells infected with nontarget shRNA, considered as calibrator. Data show the mean of three independent experiments ± SD in which samples were analyzed in technical triplicates for each condition (**p < 0.005, paired Student's *t*‐test).

On the basis of our observation of CRBN relocation at the proximity of the leading edge with partial co‐localization with F‐actin in chemokine‐treated NK cells, we tested the role of CRBN in NK cell migration. As shown in Fig. 3A and B, in primary NK and NK‐92 cells with reduced CRBN levels, the migratory ability in response to CXCL12 stimulation was significantly impaired.

Then, we evaluated the possible effects of CRBN on the ability of chemokine CXCL12 to induce the expression of a β2 integrin neoepitope that is associated with the high‐affinity state of LFA‐1 [30]. NK cells infected with lentivirus pLKO.1‐shRNA‐CRBN‐GFP or nontarget shRNA were stimulated with CXCL12 and assayed by FACS analysis for the expression of the activation‐dependent epitope of β2 integrin subunit. The results show that CRBN depletion reduced the expression of active LFA‐1 upon chemokine stimulation (Supporting Information 5A), suggesting its involvement in the regulation of chemokine induced‐outside‐in signaling.

CRBN functions as a substrate receptor for the CLR4^CRBN^ complex but can also exert ubiquitin‐independent activities [19]. To investigate whether CRBN‐associated ubiquitylation activity plays a role in these mechanisms, we infected NK‐92 cells with lentivirus expressing ROC1 shRNA, another important component of CLR4^CRBN^ complex.

Consisting with data obtained in CRBN‐depleted NK cells, we observed that ROC1‐silenced NK‐92 cells have a reduced migration toward CXCL12 (Fig. 3C and D). Moreover, similar results were obtained upon treatment of NK‐92 cells with MLN4924, a cullin E3 ligase inhibitor (Fig. 3E). These different conditions did not affect CXCR4 expression, and they were not associated with a different responsiveness of NK cells to the chemotactic stimulus (Supporting Information 6B–E).

These findings indicate that E3‐ligase activity of CRBN has an important role in the regulation of NK cell migration.

### CRBN is an upstream regulator of the small GTPase Rac1

**Figure 4:**
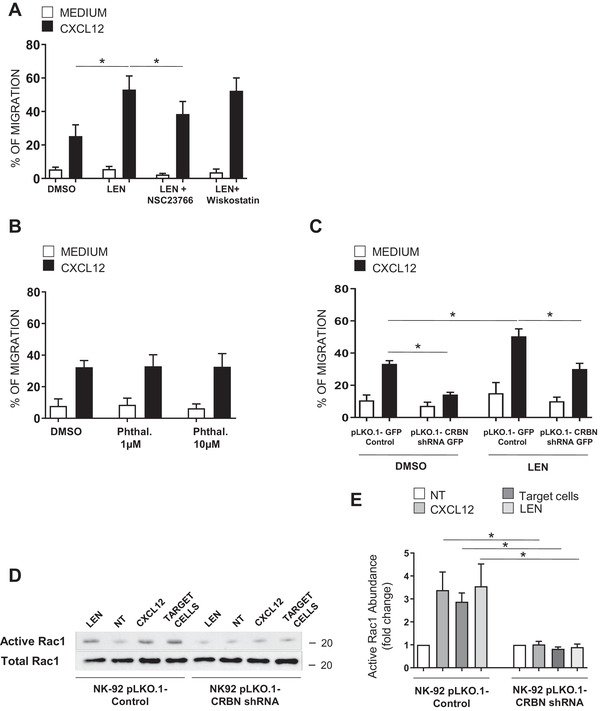
CRBN‐dependent Rac1 activation in NK cells. Primary NK cells treated with 1 μM lenalidomide (LEN) or with control vehicle (DMSO) for 18 h in the absence or presence of 0.5 μM NSC23766 or 0.5 μM wiskostatin (A) or with the indicated concentrations of phthalimide (Phthal.) (B) were assayed for their ability to migrate by flow cytometry. Results shown are the mean ± SD of the percentage of migrated cells obtained from at least three independent experiments with duplicate samples for each condition per experiment (*p < 0.05, ANOVA). (C) Migration of NK cells infected with lentivirus pLKO.1‐shRNA‐CRBN‐GFP or nontarget shRNA untreated or treated with LEN and measured by flow cytometry. The mean ± SD of the percentage of migrated cells obtained from three independent experiments is shown with duplicate samples for each condition per experiment (*p < 0.05, ANOVA). (D) Control or CRBN depleted‐NK‐92 cells were incubated with K562 target cells in an E:T ratio 1:1 or stimulated with 10nM CXCL12 for 1 min at 37°C or with 1 μM LEN for 1 h. Cells were lysed and active GTP bound was detected by pull‐down assay using GST‐fusion PAK1‐PBD. Bound Rac1 (upper panel) and total Rac1 (lower panel) were detected by western blot. Original blot picture in Supporting Information 8C. (E) Quantification of active Rac1 is expressed as fold change of the control (untreated cells). Total Rac1 level was used as loading control. The band intensity was analyzed by Image J. Results shown are the mean ± SD of three independent experiments (*p < 0.05, ANOVA).

Going insight in the molecular mechanisms, we analyzed the effects on NK cell migration of lenalidomide, a drug known to modulate the activity of CRBN. IL‐2 activated human NK cells treated for 18 h with 1 μM lenalidomide (chosen based on a dose–response experiment, Supporting Information 7A) or with control vehicle (DMSO) were assayed for their ability to migrate in response to CXCL12. Lenalidomide significantly increased the migration of IL‐2 activated NK cells (Fig. 4A) but did not alter CXCR4 levels (Supporting Information 7B). Moreover, the drug increased the expression of active LFA‐1 upon chemokine stimulation (Supporting Information 5B). Together, these results indicate that lenalidomide is able to potentiate chemokine‐induced NK cell signaling and motility.

Since Rho GTPase proteins play a pivotal role in the regulation of actin dynamics during cell migration [8], we studied the involvement of Rac1 and Cdc42 in these mechanisms. To this purpose, IL‐2‐activated NK cells were pretreated with the Rac1 inhibitor NSC23766 or with Wiskostatin, a selective inhibitor of Wasp, the effector of Cdc42, stimulated with lenalidomide, and then assayed for CXCL12‐induced migration. As shown in Fig. 4A, we did not observe any change in the presence of Wiskostatin while the Rac1 inhibitor NSC23766 could partially reverse the effect of the drug. These results indicate that modulation of small GTPase Rac1, but not Cdc42, plays a role in the regulation of NK cell migration by lenalidomide.

To address whether CRBN is involved in the regulation of NK cell migration by lenalidomide, we used two different approaches. First, we treated NK cells with phthalimide, a thalidomide analogue lacking the glutarimide moiety, which is the structural component mediating the binding to CRBN [20, 21, 22]. We did not observe any changes in chemokine‐induced migratory response of NK cells, also using doses higher than lenalidomide (Fig. 4B), thus suggesting the involvement of CRBN in these mechanisms. Second, we tested the effects of lenalidomide in CRBN‐depleted NK cells. As shown in Fig. 4C, we observed that the absence of CRBN compromises the stimulatory effect of this drug on NK cell migration.

Finally, to better dissect the molecular mechanisms underlying these effects, we examined whether the IMiDs could stimulate Rac1 activation and whether CRBN could affect the activation of this small GTPase in chemokine or target cell‐treated NK cells. To this purpose, control‐infected and CRBN‐silenced NK‐92 cell line were treated with lenalidomide, CXCL12, and K562 target cell and cell lysates were subjected to pull‐down assay using a GST‐PAK fusion protein that specifically binds to the active form of Rac1. We observed that stimulation of NK‐92 cells with lenalidomide as well as CXCL12 or target cells results in Rac1 activation. Of note, we also found that CRBN depletion significantly compromises Rac1 activation in response to the different stimuli (Fig. 4D and E), thus indicating CRBN as an upstream regulator of this small GTPase.

Together, these results indicate that CRBN‐mediated Rac1 activation plays an important role in the regulation of NK cell migration and mediates the effects of IMiDs on this process.

## Discussion

In this study, we demonstrated that CRBN is expressed in NK cells and regulates critical functions, such as migration and cytotoxicity. Increasing interest in this protein arises from its identification as the main target of IMiDs, showing important antitumor and anti‐inflammatory effects [31, 32, 33, 34]. IMiDs are known to stimulate NK cell activity, but the molecular mechanisms have been poorly investigated. By taking advantages of lenalidomide capability to bind and modify CRBN function, we demonstrated that this drug increases NK cell migration and that CRBN plays a pivotal role in these mechanisms. Intriguingly, CRBN silencing by itself significantly compromises CXCL12‐induced NK cell migratory ability, indicating that this protein takes part in chemokine signaling and it may regulate cellular processes critical for NK cell functions. Interestingly, we described for the first time that stimuli able to induce actin remodeling, including CXCL12 and target cells, cause a cellular redistribution of CRBN, which partially co‐localizes in sites of actin accumulation.

IMiDs have been shown to affect actin dynamics in monocytes and NK cells also in the absence of any other stimuli [15, 35], but the role of CRBN in these mechanisms has never been explored. CRBN shows a ubiquitous subcellular localization in unstimulated NK cells, therefore its translocation and partial overlapping with actin upon target cells/chemokine‐induced NK cell activation strongly supports a role for this protein in cytoskeleton rearrangements.

The impact of the cellular redistribution on CRBN activity was supported by the recruitment of this protein in aggresomes and its cytoprotective role in cells exposed to proteasome inhibitors [36] as well as the requirement of nuclear import of CRBN for pomalidomide‐mediated anti‐myeloma activity [37]. Indeed, these studies suggested that spatial overlap between CRBN and its substrates is important for their ubiquitination and degradation and for the efficacy of CRBN modulators.

To date, different E3 ubiquitin ligase‐dependent and independent activities for CRBN have been reported. Among immune cells, CRBN emerged mainly as a negative regulator. CRBN was found to be an important antagonist of T‐cell activation via epigenetic repression of Kv1.3 potassium channel expression [24]. Furthermore, it was also demonstrated that CRBN can negatively regulate TLR4 signaling via direct interaction with TRAF6 and TAB2 in macrophages [38]. In IMiDs‐treated NK cells, CRBN recently emerged as a key regulator of IKZF3‐dependent transcriptional repression of GZM‐B expression and cytotoxicity [16].

We propose a positive role for CRBN in NK cells, demonstrating that it is required for initial signaling events triggered by NK cell binding to target cells. Indeed, CRBN depletion was associated with a lower ability to form conjugates with target cells and less mature synapses leading to reduced NK cell killing. Previous studies described the capability of lenalidomide to rescue adhesion and motility defects in T lymphocytes from patients with CLL by targeting Rho GTPase signaling [39]. Moreover, RASGRP1‐deficient CD8^+^ T cells display migration defects that are reversed by treatment with lenalidomide via RhoA activation [40]. Herein, we demonstrated that lenalidomide increases CXCL12‐induced NK cell migration via Rac1 activation and that the absence of CRBN impairs NK cell migration and cytotoxicity, as well as Rac1 activation in response to chemokine or target cell stimulation. In line with our data, NK cells expressing a dominant‐negative Rac1 showed a decreased ability to form conjugates and polarize their granules toward the target cell [41].

Further studies are needed to investigate the molecular mechanisms underlying CRBN‐mediated Rac1 activation. E3 ubiquitin ligase activity of CLR4^CRBN^ complex appears to be important for the regulation of NK cell motility because, as CRBN depletion, also the loss of ROC1, another member of the same CRL4 ^CRBN^ complex, or treatment with the cullin E3 ligase inhibitor MLN4924, significantly compromises chemokine‐driven NK cell migration. In this regard, accumulating evidence demonstrate that a complicated network of E3 ubiquitin ligases plays important roles in cell adhesion and migration via ubiquitination of specific substrates, such as adhesion molecules, actin polymerization regulators, small GTPases, or protein kinases [42]. In particular, ubiquitination can modulate the levels of Rho GTPase and their regulators GEFs and GAPs and can also alter the interaction among these proteins. Moreover, changes in the distribution of E3 ubiquitin ligases have been associated with their regulatory functions in actin assembly [43].

In summary, our study demonstrates that the protein CRBN is implicated in the regulation of key effector functions of NK cells, such as migration and cytotoxicity, thus unrevealing new information on complex mechanisms underlying NK cell functions. Moreover, CRBN plays an important role in stimulatory effect of lenalidomide on NK cell activity shedding light on the mechanisms underlying IMiDs effects on these immune effectors.

## Materials and methods

### Cells, reagents, and antibodies

The human K562, NK‐92, and HEK293T cell lines were purchased from ATTC and were maintained at 37°C and 5% CO_2_ in medium supplemented with 10% FBS. Note that 200 U/ml human recombinant IL‐2 was added for NK‐92 cells. All cell lines were mycoplasma‐free (Mycoplasma Test Kit, Biological Industries).

Healthy donors’ peripheral blood mononuclear cells (PBMCs) were isolated by Lymphoprep (Nycomed) gradient centrifugation. NK cells were negatively selected from PBMCs using MACS human NK cell isolation kit (Miltenyi Biotec).

Lenalidomide was purchased from BioVision, Inc. (Phthalimide, Wiskostatin), and NSC23766 and MLN4924 were purchased from Merck Life Science. Homo‐PROTAC cereblon degrader 1 was purchased from Selleckchem. The following monoclonal antibodies (mAbs) were used for immunostaining: anti‐CD3/FITC, anti‐CD56/PE, anti‐CXCR4/APC, anti‐GZM‐B/FITC and anti‐GZM‐B/APC, anti‐total LFA1/APC (BD Biosciences), anti‐LFA1 clone 24 CD11‐CD18 (Hycult Biotech), and APC Goat anti‐mouse IgG (Jackson Immunoresearch Laboratories). Phalloidin‐FITC was purchased from Thermo Fisher Scientific (Waltham).

Human recombinant cytokines IL‐2 and CXCL12 were purchased from Peprotech EC.

### RNA isolation and quantitative real‐time PCR

Total RNA was extracted using RNA isolation Kit (Norgene) and used for cDNA first‐strand synthesis in a 25 μL reaction volume according to the manufacturer's protocol for M‐MLV reverse transcriptase (Promega). cDNAs were amplified in triplicate with primers for CRBN (Hs00372271_m1), GZM‐B (Hs00188051_m1), ROC1 (Hs00360274_m1), and GAPDH (Hs03929097_g1) conjugated with fluorochrome FAM (Applied Biosystems) using the ABI Prism 7900 Sequence Detection system (Applied Biosystems). The level of expression was measured using Ct (threshold cycle) as previously described [44].

### Plasmids

For knocking down CRBN in NK‐92 cell line, we used a pLKO.1‐shRNA‐CRBN (TRCN0000141562) lentiviral vector with puromycin resistance and the control vector pLKO.1 non‐targeting shRNA (MISSION Merck Life Science). For knocking down CRBN expression in primary NK cells, we used pLKO.1‐shRNA‐CRBN‐GFP‐lentivirus vector and the control vector pLKO.1 non‐targeting shRNA GFP‐lentivirus vector gently provided from Dr K.A. Stewart [45]. For knocking down ROC1 expression in NK‐92 cell line, we used LT‐virus expressing scrambled control siRNA (LT‐Control‐GFP) or siRNA targeting ROC1 (LT‐shRNAROC1‐GFP) gently provided from Dr. Yi Sun [46].

### Virus production and in vitro transduction

For lentivirus production, 5 μg of viral DNA was transfected into semi‐confluent HEK293T cells using Lipofectamine Plus (Thermo Fisher Scientific) as previously described [47]. After 72 h, virus‐containing supernatants were filtered, concentrated by ultracentrifugation at 27 000 rpm for 2 h, and used immediately for infections. Two cycles of infection were performed on IL‐2 (500 U/mL) activated 0.5 × 10^6^ NK cells in 200 μL complete medium with Polybrene (8 μg/mL) (Merck Life Science) for 2 h. For GFP‐expressing viruses, the efficiency of infection was evaluated after 3 days by measuring GFP expression using FACS analysis. After infection, NK‐92 cells were allowed to expand for 24 h and were then selected for puromycin resistance (2 μg/mL). In some experiments, NK cells infected were sorted for GFP expression at day 3 postinfection using FACSAria III (BD Bioscience) equipped with a 488 nm laser and analyzed using a FACSDiva Software (BD Bioscience version 6.1.3).

### Western blot analysis and Rac1 activation assay

For western blot analysis, total and nuclear proteins from primary NK cells were prepared as previously described [48]. Antibodies against *β*‐actin and CRBN were purchased from Merck Life Science. Antibody against lamin A, tubulin, IKZF1, and IKZF3 were purchased from Santa Cruz Biotechnology. An HRP‐conjugated secondary Ab and an ECL detection system (GE Healthcare) were used to reveal immunoreactivity following the manufacturer's instructions.

Rac1 activation assay was performed as previously described [30].

### Migration assay and chemokine‐induced expression of β2 integrin activation‐neoepitope

Purified NK cells from healthy donors or NK cells infected with lentivirus pLKO.1‐shRNA‐CRBN‐GFP or nontarget shRNA were treated for 18 h with 1 μM lenalidomide or DMSO in the presence of IL‐2 (200 U/mL) in complete medium RPMI 1640 10% FBS. Cell migration and chemokine‐induced expression of β2 integrin activation‐neoepitope was assayed as previously described [30]. The percentage of migrated cells was calculated as follows: number of migrated NK cells/number of input NK cells x 100. The percentage of migrated GFP‐positive NK cells was calculated as follows: number of GFP positive migrated NK cells/number of input GFP positive NK cells x 100.

### Immunofluorescence and flow cytometry

The surface expression of CXCR4 was analyzed on freshly isolated NK cells after treatment of 18 h with 1 μM lenalidomide or DMSO in the presence of IL‐2 (200 U/ml) or on infected cells (primary NK cells and NK‐92 cell line) by immunofluorescence staining using anti‐CXCR4/APC and mouse IgG1/APC isotype control Ab. Fluorescence was analyzed using a FACSCanto II flow cytometer.

### Conjugate assay

Scramble and CRBN silenced primary NK or NK‐92 cells were mixed with Cell Tracker Deep Red‐loaded K562 target cells at a 1:2 ratio. Cells were pelleted at 1000 g for 1 min and incubated at 37°C for different times. The cells were slowly resuspended five times and immediately fixed with 4% PFA for 30 min at room temperature. Then, cells were washed with PBS and stained with anti‐CD56/PE mAb for 20 min. Flow cytometry was used to measure conjugate frequency. Conjugates were identified as double positive cells (PE/Deep Red) on total or gated GFP positive cells for NK‐92 and primary NK cells, respectively.

### Cytotoxicity assay

A standard 4‐h chromium‐release assay using as effector cells pLKO.1‐scramble or pLKO.1‐CRBN shRNA NK‐92 cells was performed as previously described [49].

### Confocal microscopy

For chemokine stimulation, we allow primary NK cells to adhere on poly‐l‐lysine‐coated multichamber slides in the presence of CXCL12 for 30 min at 37°C. For analysis of CRBN at NK cell IS, primary NK cells or NK‐92 cells were incubated with K562 target cells in a 1:1 ratio for 7 min a 37°C. Cells were resuspended, allowed to adhere and fixed as previously described [50], stained with mouse anti‐perforin Ab, Phalloidin‐FITC, and rabbit anti‐CRBN Ab, followed by Alexa Fluor 647–conjugated goat anti‐mouse antibody and Alexa Fluor 594–conjugated goat anti‐rabbit antibody. The coverslips were mounted with SlowFade Gold reagent (Thermo Fisher Scientific), acquired with an FV1200 MPE laser‐scanning confocal microscope (Olympus Life Sciences), and processed with ImageJ software. To determine the percentage of fluorescence of CRBN at IS and the percentage of mature or immature synapse, a polarized region was defined as a triangular area with its tips located at the edge points of the NK‐target cell interface and the center of the NK cell. Granules containing perforin were considered polarized if 70% of granules were localized within this area. The amount of CRBN that localizes to the IS upon conjugation was calculated by analyzing the fluorescence intensity in the polarized region out of total CRBN fluorescence intensity in NK cells.

### Statistical analysis

Statistical significance between two groups was determined by performing two‐tailed, paired Student's *t*‐test. Differences between multiple groups were analyzed with two‐way analysis of variance (ANOVA). Prism 6 (GraphPad) software was used. Graphs show mean values, and error bars represent the SD.

## Conflict of interest

The authors declare no commercial or financial conflict of interest.

## Study approval

Human peripheral blood was obtained from donors after written informed consent and in accordance with Research Ethics Committee guidelines at the Policlinico Umberto I Hospital. All Research Ethics Committee guidelines were in accordance with the Declaration of Helsinki principles.

## Author contributions

C.F. and H.S. designed research, performed the experiments, analyzed results, and wrote the manuscript; A.K., S.R., and C.C. performed the experiments and analyzed the results; G.P. performed cell sorter experiments; A.S., A.Z., and M.C. contributed with analytic tools and analyzed the results; R.M. and R.P. performed microscopic analysis; M.C. and A.G. critically reviewed the manuscript; and A.S. contributed to design research and write the manuscript. All authors have contributed and approved the final version of the paper.

### Peer review

The peer review history for this article is available at https://publons.com/publon/10.1002/eji.202149269

**Abbreviations**
CRBN: cereblon
IS: immunological synapse
CLR4^CRBN^
: cullin‐4‐RING ubiquitin ligase
ROC1: RING‐finger protein
GZM‐B: granzyme B
IMiDs: immunomodulatory drugs
IKZF: Ikaros transcription factor

## Supporting information

Supporting Information

## Data Availability

All data that support the findings of this study are available from the corresponding author upon request.
