# Supplementary material for: Cereblon regulates NK cell cytotoxicity and migration via Rac1 activation
Source: Eur J Immunol. 2021 Sep 18;51(11):2607–17. doi: 10.1002/eji.202149269 (PMC9291148; doi:10.1002/eji.202149269)
Supplement: Supplementary file 1 — Supporting Information [file EJI-51-2607-s001.docx]

**Supporting Information**


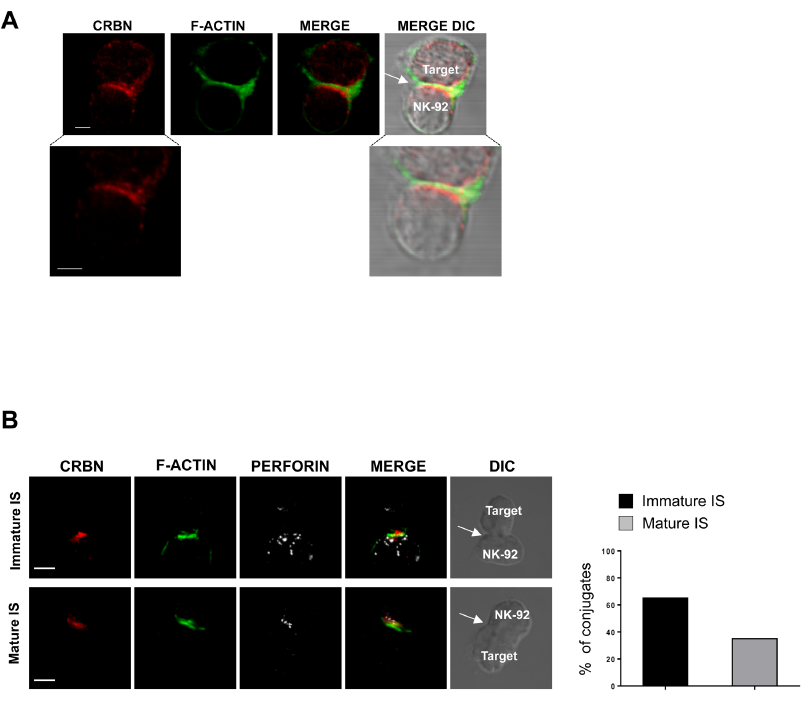


**Supporting Information 1. CRBN localizes at IS synapse in NK-92 cells. (A)** NK-92 cells were incubated with K562 target cells and conjugates were analyzed for fluorescence microscopy as described above. Fluorescence images shown were acquired using 60×/1.35 NA objective with zoom 6 (upper panels) or 18 (bottom panels). (**B)** NK-92 cells were scored for polarization of perforin toward the target cell interface. Histogram represents the percentage of conjugates with only F-Actin and CRBN (black bar, Immature IS) or also perforin (gray bar, Mature IS) at IS (right panel). The results of n≥ 30 conjugates of 2 independent experiments are shown. Scale bar represents 5μm. DIC, differential interference contrast.


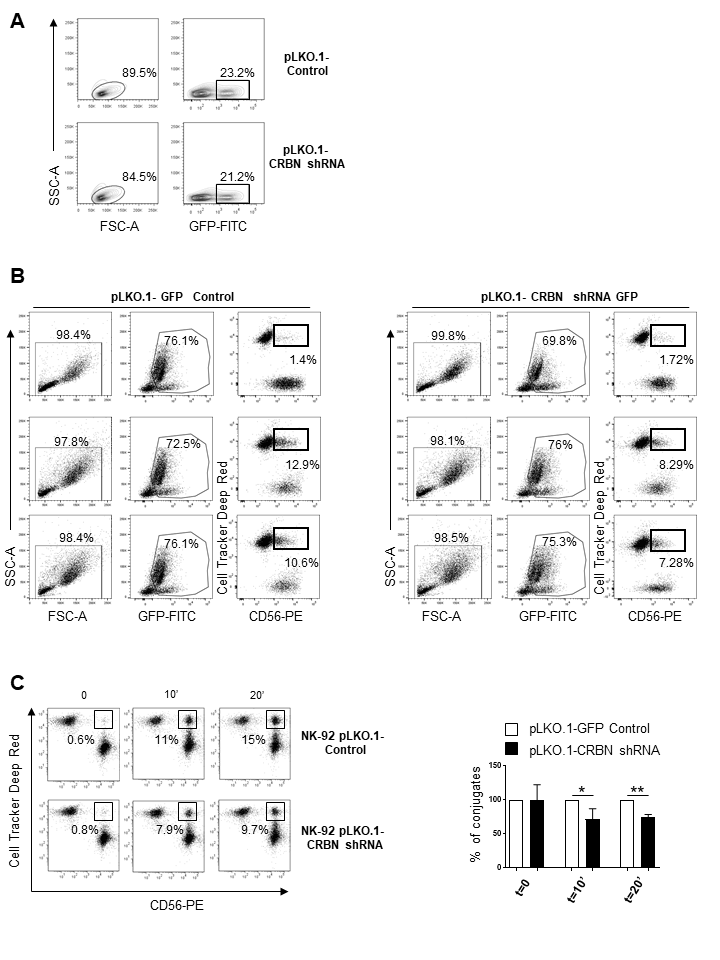


**Supporting Information 2. CRBN plays a role in conjugate formation of NK-92 with target cells. (A)** The gating strategy used to sort GFP positive cells from primary NK cells infected with pLKO.1-shRNA-CRBN-GFP or non-target shRNA (72h) to analyze CRBN mRNA expression described in Fig. 2B. (**B)** The gating strategy used to determine the percentage of conjugates of primary NK cells infected with pLKO.1-shRNA-CRBN-GFP or non-target shRNA by FACS analysis described in Fig. 2C is shown. (**C)** NK-92 cells transduced with lentivirus pLKO.1-shRNA-CRBN or non-target shRNA were incubated with Cell Tracker Deep Red-loaded K562 target cells for the indicated time, fixed and stained with anti-CD56-PE Ab. The percentage of the double positive cells (PE/Deep Red) is shown (left panel). Histogram represents the percentage of effector-target conjugates of shRNA-CRBN NK-92 cells compared to shRNA-scramble NK-92 cells, considered as 100%, at t=0, 10 and 20 min (right panel). The mean ± SD of 4 independent experiments is shown (** p<0. 005; * p<0. 05, paired Student t-test).


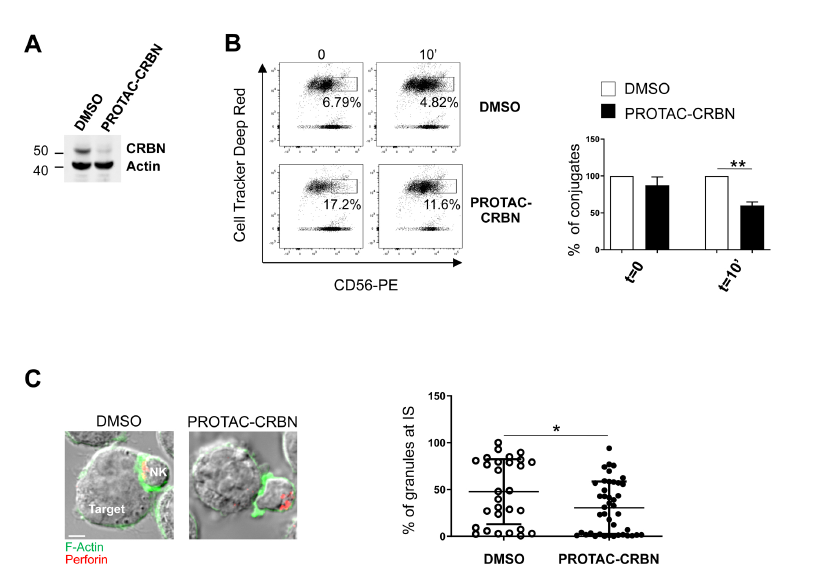


**Supporting Information 3. Impact of CRBN degradation on NK cell functions. (A)**  Western blot analysis for CRBN on total extracts obtained from primary NK cells treated with Homo-PROTAC cereblon degrader 1 (PROTAC-CRBN) (0.5 µM) or with control vehicle (DMSO) for 48h. β-Actin was used as loading control. The blot shown is representative of 3 independent experiments with NK cells obtained from 3 different healthy donors. Original picture of immunoblot are shown in Supporting Information 9A. Primary NK cells treated with PROTAC-CRBN or with control vehicle (DMSO) for 48h were incubated with K562 target cells and conjugates were analyzed by FACS analysis **(B)** or fluorescence microscopy **(C)** as described above. In (**B)** the percentage of the double positive cells is shown. Histogram represents the percentage of effector-target conjugates of PROTAC-CRBN-treated NK cells compared to untreated cells, considered as 100%, at t=0 and 10 min. The mean ± SD of 6 independent experiments is shown (** p<0. 005; paired Student t-test). In (**C)** a representative image of conjugates (left panel) (scale bar represents 5µm) and the percentage of perforin fluorescence at IS of a representative experiment and the mean percentage of conjugates with polarized granules ± SD of n ≥ 30 for each cell type of 1 out of 2 experiments (right panel) are shown (* p<0. 05, paired Student t-test).


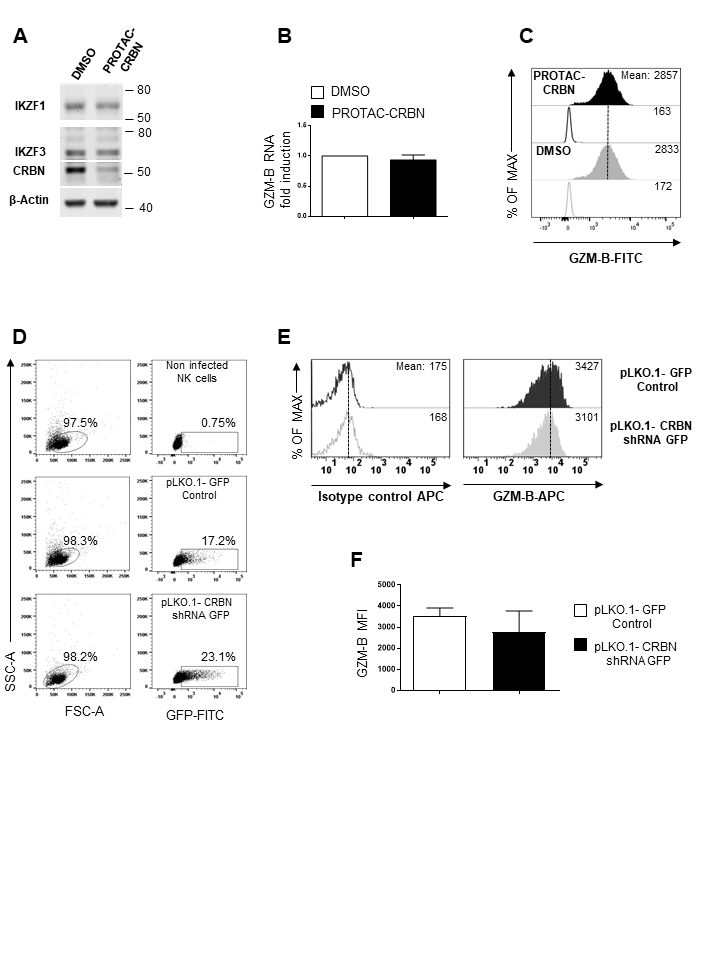
**Supporting Information 4. CRBN depletion does not affect GZM-B expression in NK cells. (A)** Western blot analysis of total extracts obtained from primary NK cells treated with Homo-PROTAC cereblon degrader 1 (PROTAC-CRBN) (0.5 µM) or with control vehicle (DMSO) for 48h. Original picture of immunoblot are shown in Supporting Information 9B. (**B)** GZM-B mRNA and protein expression was analyzed in NK cells treated with PROTAC-CRBN or with control vehicle (DMSO) for 48h by Real-time qRT-PCR (**B)** and flow cytometry (**C)**. For mRNA, results are the mean from 3 independent experiments, while for FACS 1 out of 3 experiments is shown. (**D)** IL-2 activated primary NK cells transduced with lentivirus pLKO.1-shRNA-CRBN-GFP or non-target shRNA were analyzed for GZM-B expression by FACS. The gating strategy used to determine GZM-B expression on GFP positive cells was shown (left panel). Representative data from 1 out of 3 experiments is shown (right panel, upper). Open profiles indicate isotype Ab control, while filled profiles indicate anti-GZM-B mAb. Histograms represent the MFI of specific mAb - MFI of isotype control. Data show mean ± SD calculated based on at least 3 independent experiments (paired Student t-test) (right panel, bottom).


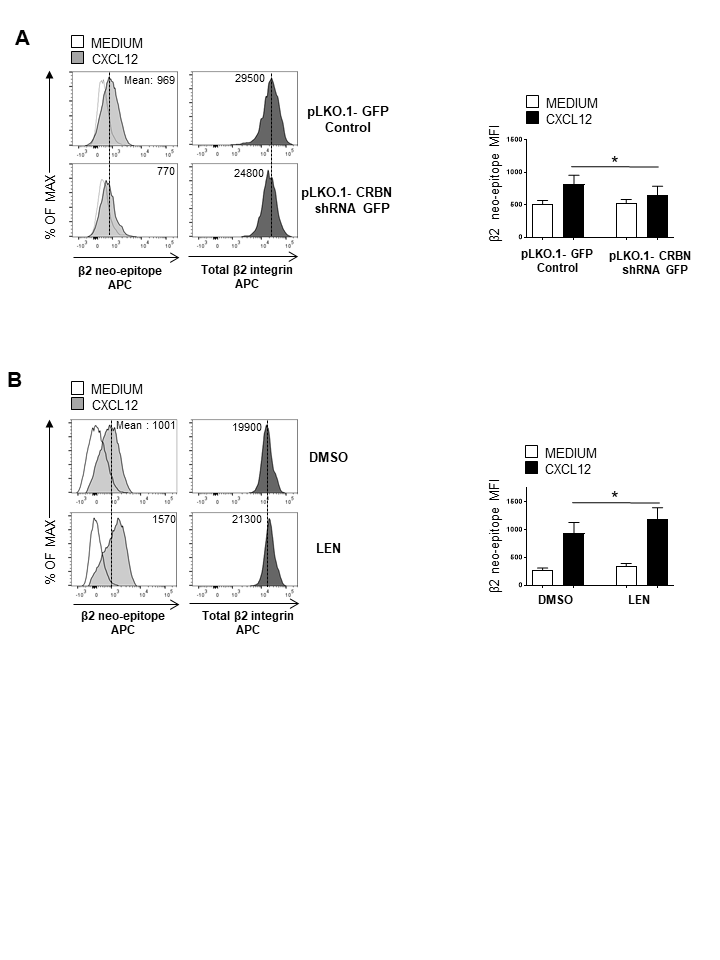
**Supporting Information 5. Effects of CRBN and Lenalidomide on chemokine-induced LFA-1 high-affinity state.** Primary NK cells transduced with lentivirus pLKO.1-shRNA-CRBN-GFP or non-target shRNA **(A)** or IL-2 activated NK cells treated with Lenalidomide (1 µM) (LEN) or with vehicle control DMSO for 18h **(B)** were left untreated or stimulated with CXCL12 (10 nM) and simultaneously stained with the high-affinity reporter mAb24 for 7 minutes at 37°C. Cells were then analyzed by flow cytometry. A representative experiment is shown (left panels). Open profiles indicate anti-β2 (mAb24) in unstimulated cells, while filled profiles indicate anti- anti-β2 (mAb24) in CXCL-12-treated NK cells. Histogram represents the mean ± SD from 3 independent experiments (**P*<0.05, ANOVA) (right panels).

**
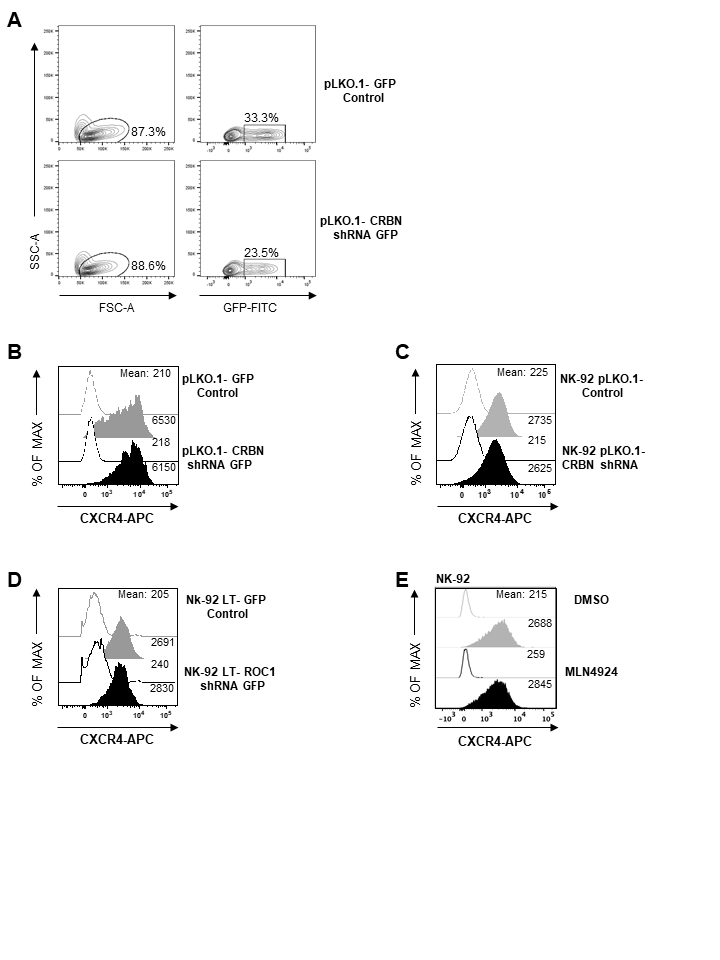
**

**Supporting Information 6.** **CXCR4 expression does not change on CRBN and ROC1-silenced NK cells**. **(A)** The gating strategy used to determine the number of migrated GFP^+^ NK cells by FACS analysis described in Fig. 3A is shown. Membrane expression of CXCR4 was analyzed on primary NK and NK-92 cells transduced with the indicated lentivirus **(A, B and D)** or treated with MLN4924 **(E)** by FACS analysis. One out of 3 experiments is shown. Open profiles indicate isotype Ab control, while filled profiles indicate anti-CXCR4 mAb.

**
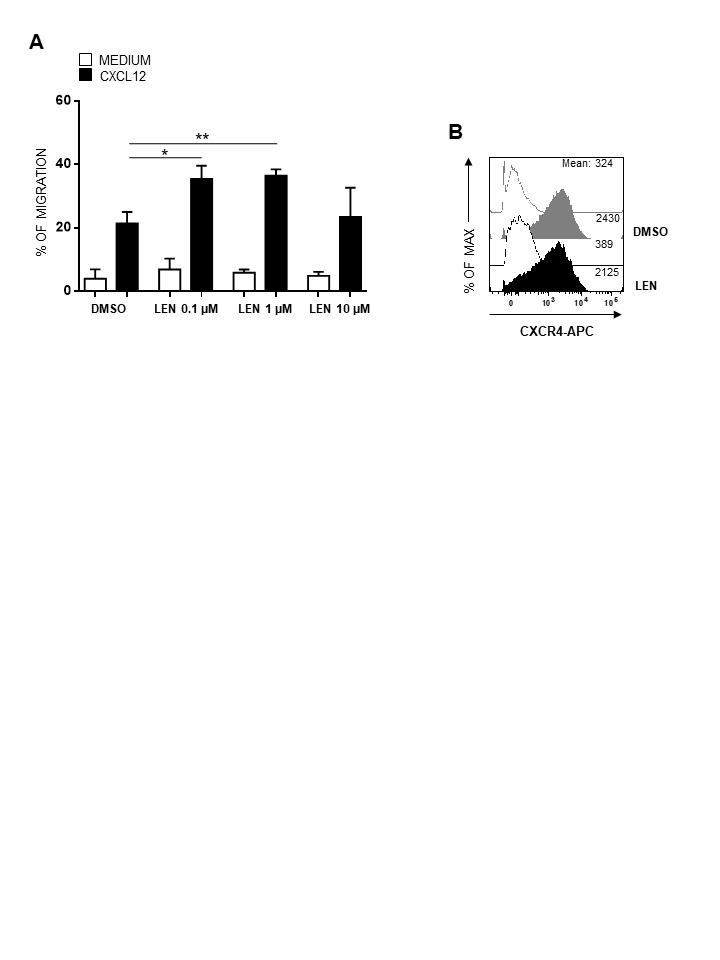
**

**Supporting Information 7. Dose-dependent effect of Lenalidomide on NK cell migration. (A)** IL-2 activated primary NK cells treated with different concentrations of Lenalidomide (LEN) (0.1, 1 and 10 µM) or with control vehicle (DMSO) for 18h were assayed for their ability to migrate through transwell towards a 10 nM CXCL12 gradient as described above. Results shown are the mean of the percentage of migrated cells obtained from at least 3 independent experiments. Error bars indicate SD (***p*< 0.005; **P*< 0.05 ANOVA). (**B)** Membrane expression of CXCR4 was analyzed on IL-2 activated-NK cells treated with 1 µM LEN or with control vehicle DMSO for 18h by FACS analysis. Representative data from 1 out of 3 experiments is shown. Open profiles indicate isotype Ab control, while filled profiles indicate anti-CXCR4 mAb.


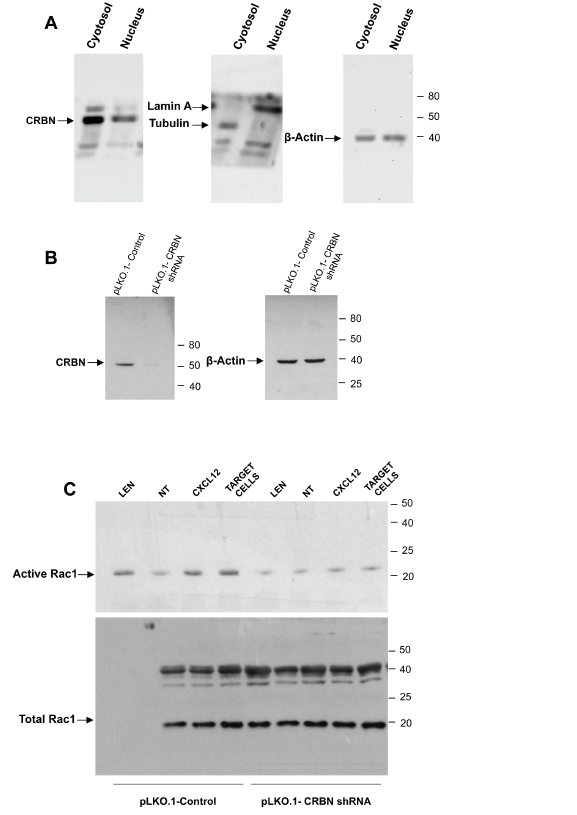


**Supporting Information 8. Western blot. (A)** Original picture of immunoblot shown in Fig. 1A. **(B)** Original picture of immunoblot shown in Fig. 2A. **(C)** Original picture of immunoblot shown in Fig. 4D.


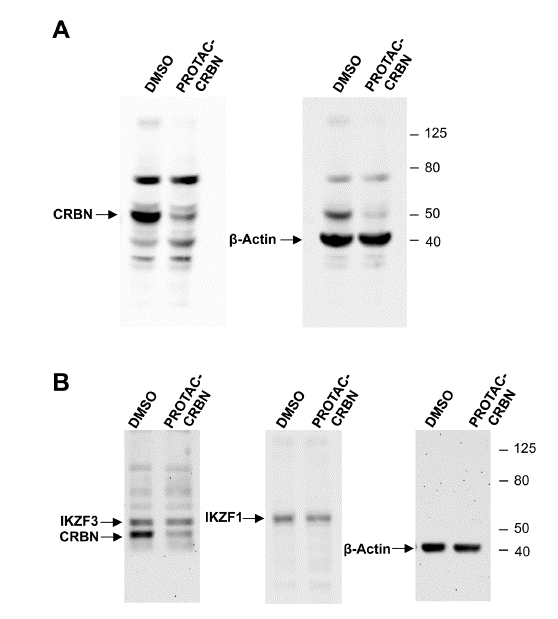


**Supporting Information 9. Western blot. (A)** Original picture of immunoblot shown in Supporting Information 3A. **(B)** Original picture of immunoblot shown in Supporting Information 4A.
